# Volume and Attenuation Characteristics of Chronic Subdural Hematoma: An Annotated Patient Cohort of 257 Patients with Interrater Reliability Assessments

**DOI:** 10.3390/tomography11120141

**Published:** 2025-12-16

**Authors:** Mattias Drake, Emma Hall, Birgitta Ramgren, Björn M. Hansen, Johan Wassélius

**Affiliations:** 1 Department of Radiology, Skåne University Hospital, 22185 Lund, Sweden; mattias.drake@skane.se (M.D.); emma.hall@med.lu.se (E.H.); birgitta.ramgren@med.lu.se (B.R.); bjorn.hansen@med.lu.se (B.M.H.); 2 Stroke Imaging Research Group, Clinical Sciences Lund (IKVL), Lund University, 22185 Lund, Sweden

**Keywords:** chronic subdural hematoma, volumetry, imaging

## Abstract

**Background:** Accurate volumetry and imaging characterization of chronic subdural hematoma (cSDH) are essential for prognostication and treatment planning, but manual assessment is time-consuming and therefore underutilized. **Methods:** We retrospectively analyzed preoperative non-contrast CT (NCCT) scans of 257 patients undergoing first-time surgery for uni- or bilateral cSDH. Hematoma volumes were measured manually using a semi-automated area-outlining tool on every second axial slice and compared with the volumes estimated through the ABC/2 formula. Hematoma attenuation patterns and components were categorized, and interrater reliability was assessed for volume, maximum diameter, and imaging features using intraclass correlation coefficients (ICCs) and Cohen’s κ. **Results:** A total of 339 hematomas were evaluated. Manual and ABC/2 volume measurements correlated strongly (R^2^ = 0.83, ICC [3, 1] = 0.90). The interrater agreement for manual volumetry was excellent (ICC [2, 1] = 0.96). Agreement was also excellent for maximum diameter (ICC [2, 1] > 0.9) and good for midline shift assessment (κ = 0.81). Agreement was moderate for the identification of fresh clots, trabeculations, and laminations (κ = 0.62–0.72) but poor for general attenuation patterns (κ = 0.44). **Conclusions:** The manual volumetry of cSDH is feasible and highly reproducible between raters of different experience levels. These results provide a robust reference standard for the validation of automated volumetry tools and support the implementation of quantitative hematoma assessment in future clinical trials and routine care.

## Simple Summary

Chronic subdural hematoma is a common condition, especially in older adults, and the accurate measurement of its size on CT scans is essential for guiding treatment decisions. In this study, we analyzed 257 patients and found that manual measurements of hematoma volume are highly reliable between different radiologists and correlate well with a simple estimation formula. These results show that precise volume measurement is both feasible and highly consistent, providing a robust reference for developing and validating future automated tools that may assist radiologists in clinical practice.

## 1. Introduction

Chronic subdural hematoma (cSDH) is among the most common neurosurgical conditions [1,2,3]. The incidence is rising due to an aging population and an increased use of antithrombotic medication [4,5]. Endovascular treatment of cSDH has become a rapidly growing field of interest owing to reports of reduced recurrence rates compared to standard treatments [6,7,8] and refined models on the underlying pathological mechanisms behind cSDH and its recurrence [9,10,11]. In addition to a mounting volume of non-randomized data [12,13,14,15], randomized controlled trials (RCTs) recently showed a lower risk of hematoma recurrence or progression leading to reoperation than surgery alone [16,17].

Several additional randomized trials are underway, and eMMA may have the potential to become the first-line treatment for cSDH with mild symptoms [18].

Neuroimaging is a cornerstone in the management of cSDH for diagnosis, prognostication, and the evaluation of treatment effect [19,20]. Non-contrast Computed Tomography (NCCT) is the most used modality due to its availability, speed, and diagnostic accuracy [19].

With the increasing focus on endovascular treatment, imaging parameters are increasingly relevant for patient selection, treatment planning, and follow-up, including early prognostication, for example, by identifying small increases in hematoma volume.

In addition to hematoma volume [21], the risk of recurrence after conventional neurosurgical treatments has also been associated with cortical atrophy [22] hematoma volume overall attenuation patterns and the presence of septations, laminations, and fresh blood components [23]. Several different radiological classifications of cSDH have been proposed for predicting recurrence risk; one of the more commonly used is the one proposed by Nakaguchi [11].

Precise cSDH volume measurements in clinical routines are cumbersome and time-consuming; therefore, surrogate measurements, such as the maximum width in coronal or transversal planes, are common substitutes in clinical practice. The ABC/2 formula has been proposed for cSDH volume assessment [24]. However, the ABC/2 formula assumes an ellipsoid shape not typical for cSDH, which is characteristically crescent-shaped; moreover, it does not include falcine or tentorial components.

Automated delineation tools have been shown to accurately measure the volume of intracerebral hemorrhages (ICHs) better than the ABC/2 formula [25,26] and may facilitate the accurate volumetry of cSDH hematomas in clinical trials and clinical routine care.

The aim of this study was to characterize a consecutive cohort of patients undergoing surgery for cSDH using detailed hematoma volumetry and imaging assessments and to evaluate the interrater reliability for manual volume measurements and attenuation features, as well as the agreement between manual volumetry and the ABC/2 formula.

## 2. Materials and Methods

### 2.1. Study Design

This is a diagnostic accuracy study on a retrospective patient population. The STROBE guidelines for reporting on cross-sectional studies were observed.

### 2.2. Study Setting

The study was conducted at the Skåne University Hospital, the only healthcare institution in the Scania region of south Sweden providing neurosurgical care, serving a population of approximately 2 million people.

### 2.3. Participant Selection

**Figure 1:**
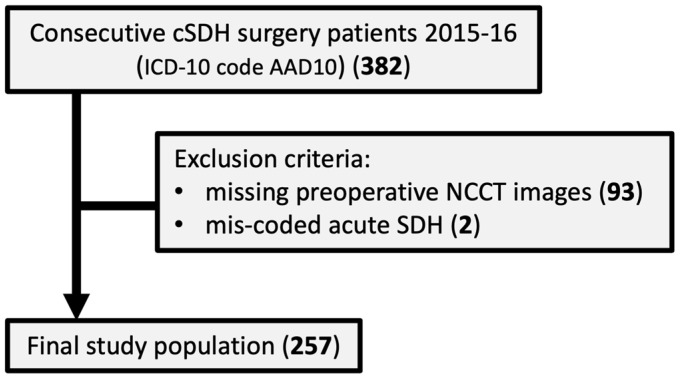
Flow chart describing the study population.

From hospital records, all patients with an ICD-10 code of AAD10 who underwent first-time surgery for uni- or bilateral CSDH during 2015 and 2016 were considered for inclusion in the study. The patient’s personal identification numbers were used to search the regional Picture Archiving and Communications System (PACS) for relevant preoperative CT examinations. The regional PACS does include the majority of hospitals in the catchment area. Patients younger than 18 years, patients with acute SDH wrongly coded, and patients without access to preoperative head NCCT images in PACS were all excluded. The final study population included 257 patients with preoperative NCCT images in relation to first-ever uni- or bilateral CSDH surgery (Figure 1).

The study was approved by the Swedish Ethical Review Authority (2024–03485–01), and individual informed consent was waived by the authority.

### 2.4. Data Collection

Patient age and sex were extracted from the Swedish personal identification number, and date of surgery was collected from the hospital medical records. These data were used to find relevant imaging in the hospital Picture Archive and Communication System (PACS).

### 2.5. Collection of Radiological Data

Preoperative NCCT images were identified for each patient. If multiple preoperative examinations were available, the examination performed closest in time before surgery was used for analysis.

### 2.6. Radiological Assessment

All imagery measurements and assessments were performed according to a prespecified reporting protocol, following assessment alignment between readers on >20 cases outside the study population.

NCCT imagery was assessed within the hospital PACS, IDS7 version v 24.2 (Sectra AB, Linköping, Sweden). Stacks of 4 or 5 mm thick reconstructed images without any overlap were generated from thin slice raw examination data. Care was taken to be as precise in delineation as possible, frequently shifting between parenchymal (center: 30/40, width: 80) and bone (center: 600, width: 2000) window settings. Where difficulties arose in separating hematoma from brain parenchyma, coronal and sagittal reconstructions and the coordinate guidance system within the IDS7 were used for increased accuracy in delineation.

### 2.7. Hematoma Volumetry

Manual volumetry was performed by outlining the hematoma borders on every second axial slice using the IDS7 area-outlining tool. Total volume was calculated by multiplying the annotated area by the slab thickness and by 2. The time to perform manual volume measurement was measured for raters 1 and 3. This measurement technique was used as a reference standard (gold standard) for subsequent volume measurements.

Volume measurement was also estimated using the ABC/2 formula (also known as the TADA formula) [27,28]. Maximum hematoma length and width on the axial slice with the largest hematoma extent were multiplied by the number of involved slices and by slice thickness, then divided by 2.

### 2.8. Hematoma Characterization

All hematomas were assessed according to the Nakaguchi classification [11]. A general hematoma attenuation pattern of hypo-, iso-, or hyperattenuating in relation to the adjacent gray brain matter was denoted. Additionally, the presence of fresh hematoma components, laminations, and trabeculations was recorded.

### 2.9. Uni- and Bilateral Hematomas

For patients with bilateral hematomas, both sides’ hematomas were treated as separate observations.

### 2.10. Radiological Assessors

One neuroradiologist with more than 8 years’ experience assessed all images with both hematoma volume measurement techniques and hematoma characterization (Reader 1). A second neuroradiologist with more than 30 years’ experience assessed 20% of images according to volume measurement 1 (Reader 2). One general radiology resident with approximately 3 years’ experience assessed the same 20% of images according to volume measurement technique 1 and hematoma characterization (Reader 3).

### 2.11. Statistical Analysis

Descriptive statistics are presented as counts (percentages, %) for categorical variables and medians (interquartile range, IQR) for continuous variables.

Agreement between manual hematoma volume measurement and the ABC/2 method for the calculation of hematoma volume was assessed using a scatter plot with linear regression and a quantile–quantile plot.

The intraclass correlation coefficients were assessed using a two-way mixed effects model. Limits of agreement were defined as the mean bias ± 1.96 SD in the Bland–Altman analyses. For categorical variables, interrater agreements were quantified using Cohen’s κ with 95% confidence intervals. The thresholds for interpretation of the interrater agreement measures (ICC and Cohen’s κ statistic) were “poor” below 0.50, “moderate” between 0.50 and 0.75, “good” between 0.75 and 0.90, and “excellent” above 0.90.

All analyses were performed in Stata/SE version 15.1. Statistical tests were two-sided and significance level was set at *p* < 0.05. Agreement analyses were performed using the *kappa* program in Stata.

## 3. Results

### 3.1. Description of Study Population

A flow chart of the study population is shown in Figure 1.

The majority of patients were men (180/257, 70.0%), and the median age was 75 years (1 standard deviation (SD) = 12.3; range 11–96).

**Figure 2:**
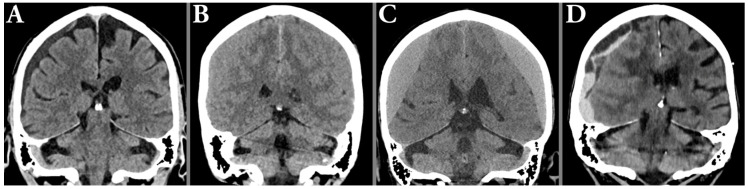
Illustration of typical radiological hematoma attenuation types; hypoattenuation (**A**), isoattenuating (**B**), hyperattenuating (**C**) in relation to the gray matter, and mixed attenuation (**D**), typically seen in acute-on-chronic hematomas.

**Figure 3:**
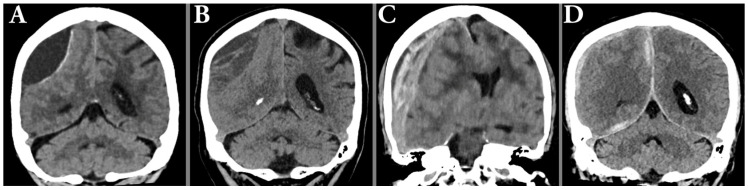
Illustration of typical radiological hematoma features such as hypoattenuating hematoma with membrane (**A**), hypoattenuating hematoma with trabeculations (**B**), laminated acute-on-chronic with midline shift (**C**), and hematoma with parafalcine and tentorial components (**D**).

Of the 257 included patients, 97 (37.7%) had left-sided hematomas, 78 (30.4%) right-sided, and 82 (31.9%) bilateral, yielding a total of 339 hematomas. Radiological characteristics of the hematomas (all, left-sided, and right-sided) are shown in Table 1, and typical radiological hematoma features are illustrated in Figure 2 and Figure 3.

### 3.2. Comparison of Manual Measurements of Hematoma Volume with the ABC/2 Method

**Figure 4:**
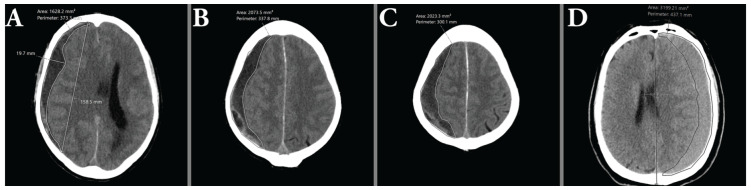
(**A**–**C**) show consecutive area measurements made on a hypoattenuating hematoma as part of the ABC/2 method. (**D**) illustrates the difficulties of measuring isoattenuating hematomas, due to the indistinct demarcation between brain parenchyma and hematoma.

An illustration of the manual volume measurements is shown in Figure 4.

**Figure 5:**
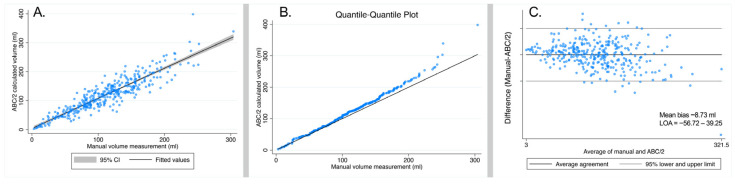
(**A**) Scatter plot of manual volume measurement compared to ABC/2 calculated volumes for 339 hematomas in 257 subjects by rater 1. (**B**) Quantile–quantile (QQ) plot of manual volume measurements compared to ABC/2 calculated volumes for 339 hematomas in 257 subjects. (**C**) Bland–Altman plot for the agreement between manual volume measurements and the ABC/2 calculated volumes of 339 hematomas in 257 subjects. The mean bias (average difference between methods and limits of agreement (LOA = bias ± 1.96 × SD)) is shown in the graph.

Figure 5A shows a scatter plot with a regression line for the manual volume measurements compared to the ABC/2 calculated volume for 339 hematomas by rater 1, and Figure 5B shows the Q-Q plot. There was a strong correlation between the manual and the ABC/2 method (beta = 0.80, 95% CI: 0.76–0.84; *p* = 6.0 × 10^−133^; R^2^ = 83.3%).

The agreement between the manual and ABC/2 calculated volume measurements (intraclass correlation using a two-way fixed effects model) showed an ICC [3, 1] of 0.90 (95% CI: 0.88–0.92; *p* < 0.0001). The Bland–Altman plot for the agreement between the manual and the ABC/2 method is shown in Figure 5C.

### 3.3. Interrater Reliability of Manual Measurements of Hematoma Volume Based on Three Independent Readers

Among the 339 hematomas, 70 hematomas were manually assessed to calculate hematoma volume by three independent raters. The median time to complete manual volume measurement was 5:47 (IQR 4:02–8:34) for rater 1 and 10:00 (IQR 9:05–12:18) for rater 3.

**Figure 6:**
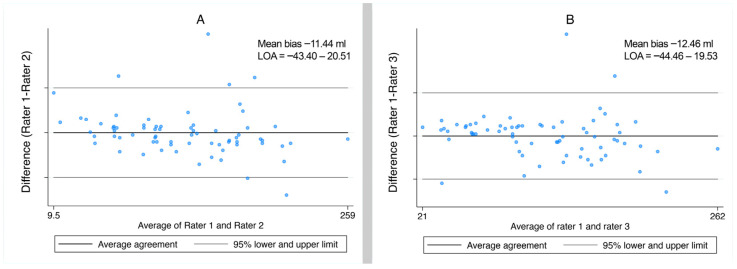
Bland–Altman plots for agreement for manual volume measurements by rater 1 compared to rater 2 (**A**) and by rater 1 compared to rater 3 (**B**). The mean bias (average difference between methods and limits of agreement (LOA = bias ± 1.96 × SD)) is shown in the graph.

The ICC [2, 1] (interrater reliability using a two-way mixed effects model) assessing the global agreement between all three raters was 0.9594 (95% CI: 0.9404–0.9732; *p* < 0.0001). Figure 6 shows the Bland–Altman plots for agreement between rater 1 compared to rater 2 and between rater 1 compared to 3, respectively.

### 3.4. Interrater Reliability of Radiological Features of Hematomas

We examined the interrater reliability of two independent raters for radiological features of CSDH. The ICC for hematoma volume and maximum diameter in the coronal plane and transversal plane showed an excellent interrater agreement (ICC [1, 3] > 0.9). For categorical variables, including general attenuation patterns, various hematoma components, and midline shift to the opposite side of the hematoma, we calculated Cohen’s kappa to assess the between-rater reliability. Among the investigated features, the general attenuation patterns showed only a poor agreement (Cohen’s kappa = 0.44, 95% CI: 0.29–0.60). The corresponding reliability measures for hematoma components, including the presence of fresh clots, trabeculations, or laminations within the hematoma, were moderate (Cohen’s kappa between 0.62 and 0.72). The best agreement was seen for the assessment of midline shift to the opposite side of the hematoma, where the interrater Cohen’s kappa value was 0.81 (95% CI: 0.69–0.92) (Table 2), indicating good agreement.

## 4. Discussion

Neuroimaging, primarily NCCT, is the imaging cornerstone in the management of cSDH for diagnosis, prognostication, and the evaluation of treatment response [19,20].

With improvements in scanner technology and image analysis tools, including AI-based tools [29,30,31], the diagnostic yield from NCCT may increase considerably and provide key imaging features that are relevant for prognostication [11,23]. Hematoma volume is of particular importance, as it is of prognostic importance in many conditions [32,33,34], as well as for CSDH [21].

Our results in this study, with a consecutively included population, a high regional high coverage, and multi-rater design, demonstrate that precise manual volume measurement is feasible and highly reproducible between readers of varying experience levels. Reliable cSDH volume measurement may be a key outcome measure in future trials, as well as in clinical routine care, to prognosticate clinical success or failure and select patients for additional follow-up or treatments, and in all these use-cases the variability of the measurement is crucial. The low interrater variability observed in this study supports the use of manual volumetry as a reliable reference standard going forward. This is encouraging, since exact manual volumetry is often perceived as being due to a similar attenuation of the brain and the hematoma.

Volumetry data from well-annotated consecutive cSDH-cohorts may also play an important role in validating automated image analysis tools for cSDH volume measurements, which are likely to become available in the near future.

Similarly, we show a high interrater agreement for the assessment of qualitative imaging features important for the prognostication of CSDH, such as the trabeculation or lamination of the hematoma.

### Limitations

This study has several limitations. First, its retrospective, single-center design and the clinically selected cohort of patients undergoing cSDH surgery may introduce selection bias and limit the generalizability of the findings. Second, although volumetric measurements were performed by multiple readers, the absence of a formal interrater agreement analysis restricts our ability to quantify observer variability. Finally, because the cohort reflects only surgically treated patients, the findings may not be fully applicable to individuals managed conservatively.

## 5. Conclusions

In this study, we demonstrate that the manual measurement of cSDH volume is both technically feasible and highly reproducible across readers with different levels of radiological experience. This consistency highlights the robustness of volumetric assessment as an objective indicator of disease burden. Beyond its methodological reliability, cSDH volume offers clinically meaningful information that surpasses traditional qualitative assessments.

## Figures and Tables

**Table 1:** Radiological characteristics of 339 chronic subdural hematomas among 257 subjects with first-time operation during 2015 and 2016.

Characteristic	All Hematomas (n = 339)	Left-Sided Hematomas (n = 179)	Right-Sided Hematomas (n = 160)
**Hematoma volume**			
Manual, mL (median, IQR)	111 (79–147)	116 (80–153)	109.5 (69.5–142.5)
ABC/2, mL (median IQR)	122 (80–155)	126 (85–156)	117.5 (70–155)
**General attenuation pattern N (%)**			
Homogenous:			
Hypo-	134 (39.5)	73 (40.8)	61 (38.1)
Iso-	40 (11.8)	21 (11.7)	19 (11.9)
Hyper-	5 (1.5)	3 (1.7)	2 (1.3)
Heterogenous:			
Diffuse pattern	125 (36.9)	62 (34.6)	63 (39.4)
Separated pattern	35 (10.3)	20 (11.2)	15 (9.4)
**Hematoma components N (%)**			
Fresh clot	209 (61.7)	113 (63.1)	96 (60.0)
Trabeculations	225 (66.4)	125 (69.8)	100 (62.5)
Laminations	37 (10.9)	22 (12.3)	15 (9.4)
**Maximum Diameter, mm (mean, SD)**			
Coronal plane	19.5 (14–24)	20 (14–25)	19 (14–23)
Transversal plane	21 (16–26)	22 (17–27)	20 (16–25)
**Midline shift to opposite side, N (%)**			
None	105 (31.0)	48 (26.8)	57 (35.6)
1–5 mm	43 (12.7)	22 (12.3)	21 (13.1)
6–10 mm	93 (27.4)	57 (31.8)	36 (22.5)
>10 mm	98 (28.9)	52 (29.1)	46 (28.8)
Parafalcine component, N (%)	15 (4.4)	9 (5.0)	6 (3.8)

**Table 2:** Agreement of radiological features of 70 hematomas assessed by two independent raters (rater 1 and 3).

Characteristic	Rater 1	Rater 3	ICC/κ (95% CI)
**Hematoma volume**			
Manual, mL (median, IQR)	115.5 (72–156)	123 (78–168)	ICC [3, 1] 0.95 (0.93–0.97)
General attenuation pattern N (%)			**κ:** 0.44 (0.29–0.60)
Homogenous:			
Hypo-	27 (38.6)	14 (20.0)	
Iso-	7 (10.0)	5 (7.1)	
Hyper-	1 (1.4)	2 (2.9)	
Heterogenous:			
Diffuse pattern	26 (37.1)	30 (42.9)	
Separated pattern	9 (12.9)	19 (27.1)	
**Hematoma components N (%)**			
Fresh clot	43 (61.4)	29 (41.4)	**κ:** 0.62 (0.44–0.79)
Trabeculations	49 (30.0)	51 (72.9)	**κ:** 0.72 (0.54–0.91)
Laminations	9 (12.9)	5 (7.1)	**κ:** 0.69 (0.39–0.98)
**Maximum diameter, mm (median, IQR)**			
Coronal plane	19 (16–24)	19 (17–25)	ICC [3, 1] 0.94 (0.90–0.96)
Transversal plane	21 (18–27)	23 (18–27)	ICC [3, 1] 0.92 (0.87–0.95)
Midline shift to opposite side, N (%)			**κ:** 0.81 (0.69–0.92)
None	21 (30.0)	19 (27.1)	
1–5 mm	11 (15.7)	12 (17.1)	
6–10 mm	15 (21.4)	17 (24.3)	
>10 mm	23 (32.9)	22 (31.4)	

ICC = intraclass correlation coefficient, κ = Cohen’s kappa.

## Data Availability

The data presented in this study are available on reasonable request from the corresponding author. The data are not publicly available due to privacy and ethical restrictions.

## Abbreviations

The following abbreviations are used in this manuscript:

cSDH	Chronic subdural hematoma
eMMA	Embolization of the Middle Meningeal Artery
ICH	Intracerebral hemorrhage
ICC	Intraclass correlation coefficients
IQR	Interquartile range
NCCT	Non-contrast CT
PACS	Picture Archiving and Communications System
QQ plot	Quantile–quantile plot
SDH	Subdural hematoma
